# Procedure-specific outcomes of robotic versus laparoscopic Roux-en-Y gastric bypass and biliopancreatic diversion with duodenal switch across a decade of MBSAQIP adoption

**DOI:** 10.1007/s11701-026-03865-w

**Published:** 2026-09-04

**Authors:** Mohamed Hany, Mohamed H. Zidan, Ala Wafa, Ehab Elmongui, Hazem Al-Momani, Khaled Hamdan, Mahmoud Diaa Hindawi, Rodolfo Oviedo

**Affiliations:** 1 Department of Experimental and Clinical Surgery, Medical Research Institute, Alexandria University, Alexandria, Egypt; 2 Bariatric Surgery Unit, Madina Women’s Hospital, Alexandria, Egypt; 3 Alexandria University, Alexandria, Egypt; 4 The Research Papyrus Lab, Alexandria, Egypt; 5 Misurata University, Jamahiriya, Libya; 6 Independent Biostatistical Consultant, Alexandria, Egypt; 7 NMC Royal Khalifa Hospital, Abu Dhabi, United Arab Emirates; 8 Khalifa University, Abu Dhabi, UAE; 9 Health Point Hospital, Abu Dhabi, UAE; 10 Faculty of Medicine, Al-Azhar University, Cairo, Egypt; 11 Department of Surgery, Nacogdoches Medical Center, Nacogdoches, TX USA; 12 University of Houston Tilman J. Fertitta Family College of Medicine, Houston, TX USA; 13 Sam Houston State University College of Osteopathic Medicine, Conroe, TX USA

**Keywords:** Robotic surgery, Metabolic and bariatric surgery, Roux-en-Y gastric bypass, Biliopancreatic diversion with duodenal switch, MBSAQIP, Comparative effectiveness

## Abstract

Robotic adoption in metabolic and bariatric surgery has increased, but its procedure-specific short-term outcomes remain uncertain. This study compared robotic and laparoscopic Roux-en-Y gastric bypass (RYGB) and biliopancreatic diversion with duodenal switch (BPD-DS) over a decade. The Metabolic and Bariatric Surgery Accreditation and Quality Improvement Program database was queried for primary RYGB and BPD-DS performed from 2015 to 2024. Missing data were addressed using multiple imputation followed by stabilized inverse probability of treatment weighting. Propensity-score and outcome models were fitted separately within four predefined procedure-period strata: RYGB 2015–2019, RYGB 2020–2024, BPD-DS 2015–2019, and BPD-DS 2020–2024. Thirty-day outcomes were estimated within each stratum, with Benjamini-Hochberg false-discovery-rate adjustment applied across the modeled comparisons. The analysis included 411,155 patients: 87,028 R-RYGB, 307,420 L-RYGB, 5,283 R-BPD-DS, and 11,424 L-BPD-DS. Robotic utilization increased from 8.6% to 44.0% for RYGB and from 28.2% to 50.3% for BPD-DS. For RYGB, the robotic approach was associated with higher readmission in both periods (OR 1.23 and 1.19), lower odds of serious complications (OR 0.84 and 0.87), and longer operative duration (mean differences 39.83 and 26.17 min). For BPD-DS, robotic surgery was associated with higher readmission in both periods (OR 1.38 and 1.25), higher intervention in 2015–2019 (OR 1.65), and longer operative duration (mean differences 87.49 and 40.85 min). BPD-DS estimates were less precise, and some associations attenuated in the complete-case sensitivity analysis. Robotic utilization increased substantially but was associated with longer operative duration and higher readmission for both procedures, without a clear overall 30-day safety advantage. BPD-DS findings were less precise and should be considered exploratory.

**Supplementary Information:**

The online version contains supplementary material available at https://doi.org/10.1007/s11701-026-03865-w.

## Key points

Robotic utilization increased from 8.6% to 44.0% for Roux-en-Y gastric bypass and from 28.2% to 50.3% for biliopancreatic diversion with duodenal switch between 2015 and 2024.

Across both prespecified five-year coding eras, robotic surgery was associated with longer operative duration and higher 30-day readmission for both procedures.

Robotic Roux-en-Y gastric bypass showed lower odds of serious complications but higher readmission and longer operative duration, resulting in a mixed short-term outcome profile; the less precise BPD-DS findings should be considered exploratory.

**Supplementary Information:**

The online version contains supplementary material available at https://doi.org/10.1007/s11701-026-03865-w.

## Introduction

Metabolic and bariatric surgery (MBS) is the most effective durable treatment for obesity and its associated metabolic complications, with guidelines supporting its role across a broad range of body mass index (BMI) thresholds and obesity-related disease profiles [1–3]. Global registry and survey data confirm that minimally invasive MBS now dominates contemporary practice, with sleeve gastrectomy representing the most common primary procedure worldwide, while Roux-en-Y gastric bypass (RYGB) remains a major primary and revisional metabolic operation [4, 5]. In parallel with these shifts in procedure selection, robotic-assisted surgery has been increasingly adopted in MBS practice, largely because of proposed technical advantages such as three-dimensional visualization, wristed instrumentation, improved dexterity, tremor filtration, and improved surgeon ergonomics [6, 7]. Whether these technical advantages translate into superior short-term clinical outcomes remains to be proven.

RYGB and biliopancreatic diversion with duodenal switch (BPD-DS) are both anastomotic MBS procedures, but they should not be interpreted as clinically interchangeable operations. RYGB is one of the most established metabolic operations, with broad use across primary and revisional bariatric practice. BPD-DS, by contrast, is performed less frequently, mainly because it is technically more complex, and is often selected for patients in whom greater metabolic or weight-loss effect is desired. Comparative long-term evidence suggests that duodenal switch may produce greater weight and body mass index reduction than RYGB in patients with high BMI, but this benefit may come at the cost of a less favorable risk profile, including nutritional and gastrointestinal adverse effects [8, 9]. These differences in indication, anatomy, technical complexity, and expected perioperative course make it inappropriate to draw a single generalized conclusion for “gastric bypass procedures.” Robotic and laparoscopic approaches should therefore be evaluated within each procedure separately.

The comparative safety of robotic versus laparoscopic MBS remains debated. Earlier MBSAQIP-based analyses comparing robotic-assisted and laparoscopic MBS procedures reported mixed findings, including longer operative duration for robotic surgery and variable associations with postoperative morbidity [10]. More recent registry studies and systematic reviews have suggested that robotic and laparoscopic gastric bypass may have broadly comparable short-term outcomes, although robotic surgery has not consistently demonstrated a clear reduction in overall complications and may be associated with longer operative duration or selected adverse outcome signals [6, 7, 11]. In a systematic review and meta-analysis of robotic versus laparoscopic gastric bypass, robotic surgery was not associated with a significant reduction in overall complications and was associated with a slightly higher reoperation rate [11]. Similarly, contemporary MBSAQIP analyses have suggested that the relationship between robotic approach and outcomes may vary by procedure type, patient complexity, and operative context rather than reflecting a uniform platform-level benefit [6, 12].

However, these published studies have several drawbacks. First, many available studies cover shorter or earlier time windows and may not reflect the most recent expansion of robotic bariatric adoption. Second, some analyses combine different bariatric operations, limiting procedure-specific interpretation. Third, relatively few studies formally evaluate whether associations between robotic approach and short-term outcomes have changed over time as adoption increased, team experience accumulated, and perioperative pathways evolved. This is particularly relevant for RYGB and BPD-DS because their learning curves, operative demands, and complication profiles differ. A more robust analysis of data over a longer period of time is therefore needed in order to allow robotic adoption and short-term outcomes to be examined across both early and contemporary periods, while preserving procedure-specific interpretation.

Therefore, this study aimed to evaluate temporal trends in robotic utilization and procedure-specific 30-day outcomes for robotic versus laparoscopic RYGB and BPD-DS using the MBSAQIP registry from 2015 to 2024. Specifically, we compared robotic RYGB (R-RYGB) with laparoscopic RYGB (L-RYGB), and robotic BPD-DS (R-BPD-DS) with laparoscopic BPD-DS (L-BPD-DS), within two prespecified registry-coding eras (2015–2019 and 2020–2024). We hypothesized that robotic utilization would increase for both procedures and evaluated short-term outcome associations separately within each procedure-period stratum.

## Methods

### Study design and reporting

This was a retrospective comparative-effectiveness analysis of prospectively collected routinely recorded registry data from the Metabolic and Bariatric Surgery Accreditation and Quality Improvement Program (MBSAQIP) Participant Use Files. The study evaluated temporal trends in robotic adoption and procedure-specific 30-day outcomes among patients undergoing primary Roux-en-Y gastric bypass (RYGB) or biliopancreatic diversion with duodenal switch (BPD-DS) between 2015 and 2024.

The analysis was designed to compare robotic and laparoscopic approaches within each procedure rather than estimate a single pooled effect across anatomically and technically distinct operations. Accordingly, the principal comparisons were robotic RYGB (R-RYGB) versus laparoscopic RYGB (L-RYGB) and robotic BPD-DS (R-BPD-DS) versus laparoscopic BPD-DS (L-BPD-DS). Propensity-score and outcome models were fitted separately within four predefined procedure-period strata: RYGB 2015–2019, RYGB 2020–2024, BPD-DS 2015–2019, and BPD-DS 2020–2024, thereby allowing covariate balance and treatment associations to be evaluated directly within each clinically relevant stratum.

The study was reported in accordance with the Reporting of studies Conducted using Observational Routinely collected health Data (RECORD) statement, an extension of the Strengthening the Reporting of Observational Studies in Epidemiology (STROBE) guideline for studies using routinely collected health data [13].

### Target-trial framework

A target-trial framework was used to clarify the causal question addressed by this registry-based analysis. The hypothetical trial would enroll patients undergoing primary RYGB or primary BPD-DS during the study period and assign them, at the time of the index operation, to either a robotic-assisted or laparoscopic operative approach within the same procedure type. Thus, the two procedure-specific treatment contrasts of interest were R-RYGB versus L-RYGB among patients undergoing RYGB, and R-BPD-DS versus L-BPD-DS among patients undergoing BPD-DS.

Eligibility in the target trial includes patients undergoing primary procedures, excluding revisional or conversional MBS operations. Time zero is the date of the index operation. Treatment assignments are based on the recorded operative approach, with cases converting to open surgery classified separately as a postoperative or perioperative outcome.

The follow-up period lasts 30 days post-index procedure, aligning with the MBSAQIP outcome window. Outcomes of interest include 30-day unplanned reoperation, readmission, intervention, mortality, conversion to open surgery, complications, operative duration, and length of stay.

The primary aim is to assess the association between robotic-assisted and laparoscopic approaches and 30-day outcomes. A secondary objective explores differences in these associations between the early adoption period (2015–2019) and the contemporary period (2020–2024). Stabilized inverse probability of treatment weighting balances demographic, clinical, laboratory, procedural, and temporal covariates between groups. The estimates reflect adjusted observational associations under the assumptions of no unmeasured confounding, positivity, correct model specification, and accurate outcome assessment.

### Data source, setting, and ethics

Data were obtained from the MBSAQIP Participant Use Files for the years 2015 through 2024. MBSAQIP is a North American bariatric surgery quality-improvement registry that collects standardized perioperative and 30-day postoperative data from accredited MBS centers. The registry includes patient-level demographic, comorbidity, operative, perioperative, and postoperative outcome variables, with outcomes recorded within 30 days of the index operation.

The study setting was MBSAQIP-accredited centers in the United States and Canada. Because the Participant Use Files contain de-identified patient-level data, this analysis did not constitute human-subjects research, and institutional review board approval and informed consent were not required. The MBSAQIP participating centers are the source of the data, but they were not involved in the design or conduct of the present analysis and are not responsible for the statistical analyses, interpretation, or conclusions of this study.

### Study population and case selection

Annual MBSAQIP Participant Use File datasets from 2015 to 2024 were queried to identify patients undergoing primary RYGB or primary BPD-DS. RYGB procedures were identified using the principal operative procedure Current Procedural Terminology (CPT) code 43,644, whereas BPD-DS procedures were identified using CPT code 43,845. Because the BPD-DS cohort was defined using CPT 43,845, it operationally represents classic biliopancreatic diversion with duodenal switch. SADI-S/SIPS procedures were not intentionally included as separate procedure categories and would only be captured if recorded under CPT 43,845.

Because MBSAQIP variable structure changed during the study period, primary procedures were identified using year-specific case-selection logic. For the 2015–2019 datasets, revisional and conversional procedures were excluded using the CPTUNLISTED_REVCONV variable, retaining only cases coded as non-revisional and non-conversional. For the 2020–2024 datasets, primary procedures were selected by retaining only cases with PROCEDURE_TYPE coded as “Initial.” This approach was used to harmonize primary-case identification across the full study period.

Eligible cases were retained if they underwent primary RYGB or primary BPD-DS through a minimally invasive approach classifiable as either laparoscopic or robotic-assisted. RYGB and BPD-DS were retained as distinct procedure groups throughout the analysis. Cases were not interpreted as a single pooled “gastric bypass” cohort for clinical inference; rather, the analytic framework was constructed to estimate procedure-specific comparisons of R-RYGB versus L-RYGB and R-BPD-DS versus L-BPD-DS.

Patients without available 30-day follow-up data were excluded from outcome analyses. Additional exclusions were applied during data harmonization and missing-data handling as described below, including recoding implausible zero values for age, body mass index, albumin, and hematocrit as missing before imputation.

### Exposure definition: operative approach

The exposure of interest was operative approach within each procedure type. For RYGB, patients were classified as undergoing robotic Roux-en-Y gastric bypass (R-RYGB) or laparoscopic Roux-en-Y gastric bypass (L-RYGB). For BPD-DS, patients were classified as undergoing robotic biliopancreatic diversion with duodenal switch (R-BPD-DS) or laparoscopic biliopancreatic diversion with duodenal switch (L-BPD-DS).

Operative-approach coding was harmonized across MBSAQIP data years because the structure of approach variables changed during the study period. In the 2015–2019 Participant Use Files, operative approach was recorded using the principal operative approach variable. Cases coded as robotic-assisted were classified as robotic procedures. Cases coded as conventional laparoscopic were classified as laparoscopic procedures for manuscript terminology. In the 2020–2024 files, operative approach was determined using the procedural approach variable together with the robotic-assist indicator. Cases recorded as laparoscopic with robotic assist were classified as robotic procedures, whereas cases recorded as laparoscopic without robotic assist were classified as laparoscopic procedures.

The term “conventional” was not used to describe the comparator group in the manuscript, except when referring to original registry coding where necessary. Instead, the comparator was described as laparoscopic throughout. Accordingly, all procedure-specific comparisons were expressed as R-RYGB versus L-RYGB and R-BPD-DS versus L-BPD-DS.

Cases converted to an open approach were retained and analyzed according to their initial minimally invasive approach, consistent with the target-trial intention-to-treat analogue.

### Study period definition

Calendar year was categorized a priori into two periods: 2015–2019 and 2020–2024. The boundary was selected primarily because the structure and coding of the MBSAQIP operative-approach and procedure-type variables changed beginning with the 2020 Participant Use File. Different year-specific algorithms were therefore required to identify primary procedures and harmonize operative approach across the two periods.

Accordingly, these intervals should be considered two analytically harmonized registry-coding eras rather than periods separated by a statistically identified change point in robotic adoption or outcomes. The period variable was used descriptively to characterize utilization patterns and analytically to compare the association between operative approach and 30-day outcomes across the two prespecified coding eras.

No attempt was made to estimate an exact calendar year at which the association between operative approach and outcomes changed. Year-specific interaction estimates would have been statistically unstable for infrequent outcomes, particularly within the smaller BPD-DS cohort, and the coding transition itself limited the validity of a data-driven annual change-point analysis.

### Outcomes and covariates

All outcomes were assessed within the 30-day MBSAQIP postoperative follow-up window. The analyzed 30-day endpoints included mortality, prolonged index stay, unplanned readmission, unplanned reoperation, unplanned intervention, and serious complications. Prolonged index stay was defined as index hospital stay of 3 days or longer. Serious complications were defined as a composite endpoint of postoperative sepsis, septic shock, organ-space surgical-site infection, deep incisional surgical-site infection, transfusion or bleeding, pulmonary embolism, venous thrombosis requiring therapy, pneumonia, ventilator use, acute renal failure, progressive renal insufficiency, cardiac arrest requiring cardiopulmonary resuscitation, myocardial infarction, stroke. Operative length, measured in minutes, was evaluated as a continuous perioperative outcome.

Covariates were selected a priori based on clinical relevance and potential association with operative approach and postoperative outcomes. These included demographic characteristics, baseline clinical status, comorbidities, laboratory values, and selected procedural variables. Specifically, the propensity-score models incorporated age, sex, race, body mass index, American Society of Anesthesiologists physical status classification, functional status, albumin, hematocrit, diabetes, hypertension, hyperlipidemia, gastroesophageal reflux disease, chronic obstructive pulmonary disease, obstructive sleep apnea, therapeutic anticoagulation, cardiovascular and thromboembolic history, renal disease, smoking status, previous surgery, drain placement, anastomosis leak test, and documented stapler use/anastomosis completion. Propensity-score models were fitted separately within each procedure-period stratum; therefore, procedure type and study period defined the analytic strata and were not included as covariates in the stratum-specific models.

### Data harmonization and missing data

Annual MBSAQIP datasets were harmonized before merging to account for changes in variable names, coding schemes, and category structures across data years. Variables representing equivalent clinical constructs were standardized into unified formats, including diabetes status, American Society of Anesthesiologists class, sex, race, functional status, discharge disposition, conversion status, and binary outcome indicators. Implausible zero values for age, body mass index, albumin, and hematocrit were recoded as missing before analysis. After harmonization, yearly datasets were merged using shared standardized variables, and indicators for operative year and study period were generated.

Missingness was assessed for all baseline and procedural variables and examined by treatment group. Variables with minimal missingness were handled using complete-case exclusion before imputation. Albumin, hematocrit, and race were selected for multiple imputation because they had the highest proportions of missing data. Associations between missingness indicators and observed baseline, procedural, treatment, and outcome variables were assessed to evaluate the missing-data mechanism.

Multiple imputation by chained equations was performed for albumin, hematocrit, and race. Predictive mean matching was used for continuous variables, and multinomial logistic regression was used for race. The imputation model included operative approach, operative year, baseline comorbidities, procedural characteristics, and postoperative outcomes. A complete-case cohort with complete albumin, hematocrit, and race data was retained for sensitivity analysis.

### Descriptive trend analysis

Annual descriptive trend analyses were conducted to evaluate temporal changes in the utilization of robotic and laparoscopic approaches within each gastric bypass procedure type. For both RYGB and BPD-DS cohorts, annual counts and proportions of robotic versus laparoscopic procedures were calculated from 2015 to 2024 to characterize evolving surgical adoption patterns over time. The average annual relative change in the annual approach-specific proportion was calculated as [(proportion in 2024 / proportion in 2015)^(1/9) − 1] × 100, where 9 represents the number of yearly intervals between 2015 and 2024. Temporal trends in annual procedural proportions were assessed separately within each procedure type and surgical approach using weighted linear regression models with annual proportion as the dependent variable, calendar year as the independent variable, and annual procedure volume as the weight. The p values from these models were used to evaluate evidence of temporal trend and were reported separately from the average annual relative change.

### Propensity-score weighting

To minimize baseline and procedural imbalances between robotic and laparoscopic procedures, stabilized inverse probability of treatment weighting (sIPTW) was applied within each of the five imputed datasets. Propensity scores were estimated separately within each procedure-period stratum: RYGB 2015–2019, RYGB 2020–2024, BPD-DS 2015–2019, and BPD-DS 2020–2024. Within each stratum, propensity scores were estimated using generalized boosted regression models through the twang package in R. Models were specified to estimate the average treatment effect (ATE) and were constructed using 500 regression trees, an interaction depth of 2, a shrinkage rate of 0.01, and the es.mean stopping criterion, while the bag fraction and minimum terminal node size were predefined according to the analytic framework.

The stratum-specific propensity-score models incorporated demographic characteristics, baseline comorbidities, laboratory parameters, functional status, and operative variables. Included covariates comprised age, sex, race, body mass index, albumin, hematocrit, ASA classification, functional status, diabetes, hypertension, hyperlipidemia, gastroesophageal reflux disease, therapeutic anticoagulation, chronic obstructive pulmonary disease, obstructive sleep apnea, prior myocardial infarction, percutaneous coronary intervention, previous cardiac surgery, pulmonary embolism history, deep vein thrombosis history, venous stasis, dialysis, renal insufficiency, smoking status, previous surgery, drain placement, anastomosis checked, and documented stapler use/anastomosis completion.

Balance diagnostics were assessed using absolute standardized mean differences (aSMDs), with values below 0.10 considered indicative of acceptable covariate balance, and were evaluated graphically using love plots for each imputed dataset. Following stratum-specific sIPTW, all covariates achieved acceptable balance across the procedure-period strata in the first imputed dataset, with similar balance patterns across the remaining imputed datasets. Stabilized weight distributions demonstrated good numerical stability without evidence of excessive weight inflation or marked lack of overlap (Supplementary Table S3). Across imputations, mean stabilized weights remained close to unity in laparoscopic cases and ranged from approximately 0.94 to 0.99 in robotic cases. The 99th percentile of weights remained modest across strata, and maximum weights ranged from 1.15 to 2.58, supporting adequate overlap and indicating that truncation of stabilized weights was not necessary. Effective sample sizes were lower than nominal sample sizes among robotic cases across strata, reflecting the expected precision cost of weighting. The same stratum-specific weighting strategy was reproduced in the complete-case cohort as part of the sensitivity analyses.

### Outcome analysis

Outcomes were evaluated after stratum-specific stabilized inverse probability of treatment weighting. In the primary multiply imputed analysis, weighted outcome models were fitted separately within each imputed dataset and within each procedure-period stratum: RYGB 2015–2019, RYGB 2020–2024, BPD-DS 2015–2019, and BPD-DS 2020–2024. Pooled estimates were subsequently derived across imputations using Rubin’s rules.

Binary outcomes were analyzed using weighted logistic regression models and included mortality, prolonged index stay, unplanned readmission, unplanned reoperation, unplanned intervention, and serious complications. Effect estimates for binary outcomes were reported as odds ratios (ORs) with corresponding 95% confidence intervals (CIs). Operative length, measured in minutes, was analyzed as a continuous perioperative outcome using weighted linear regression models and summarized as mean differences with 95% CIs.

Procedure- and period-specific treatment effects were estimated directly within each stratum. The procedure types and two five-year coding eras were predefined; however, the revised analysis did not fit a pooled approach × procedure × period interaction term. Accordingly, between-stratum patterns were interpreted descriptively and were not considered formal tests of effect modification. To account for multiplicity, Benjamini-Hochberg false-discovery-rate adjusted p values were calculated across the full family of 28 procedure-period endpoint comparisons. The same stratum-specific analytical framework was repeated in the complete-case cohort as a sensitivity analysis.

All statistical analyses were performed using R software (R Foundation for Statistical Computing, Vienna, Austria), version 4.4.2.

## Results

### Cohort assembly and missing data

The initial analytic cohort included 431,881 patients undergoing primary RYGB or BPD-DS during the study period, of whom 416,857 (97.0%) had complete 30-day follow-up data. After exclusion of observations with minimal missingness in baseline and procedural variables, 411,509 patients were eligible for multiple imputations. Albumin, race, and hematocrit had the highest proportions of missing data and were therefore selected for imputation. Missingness was not compatible with a missing-completely-at-random mechanism, as missingness indicators were associated with several observed baseline, procedural, treatment, and outcome variables.

Multiple imputation by chained equations was performed for albumin, hematocrit, and race. After imputation, 354 observations with residual missingness were excluded, yielding 411,155 patients in each completed imputed dataset. A complete-case cohort of 288,038 patients with complete albumin, hematocrit, and race data was retained for sensitivity analysis. Detailed missingness patterns, assessment of missing-data assumptions, and imputation diagnostics are presented in Supplementary Tables S1–S2 and Supplementary Figure S1.

### Temporal trends in the utilization of robotic versus laparoscopic approaches

**Fig. 1:**
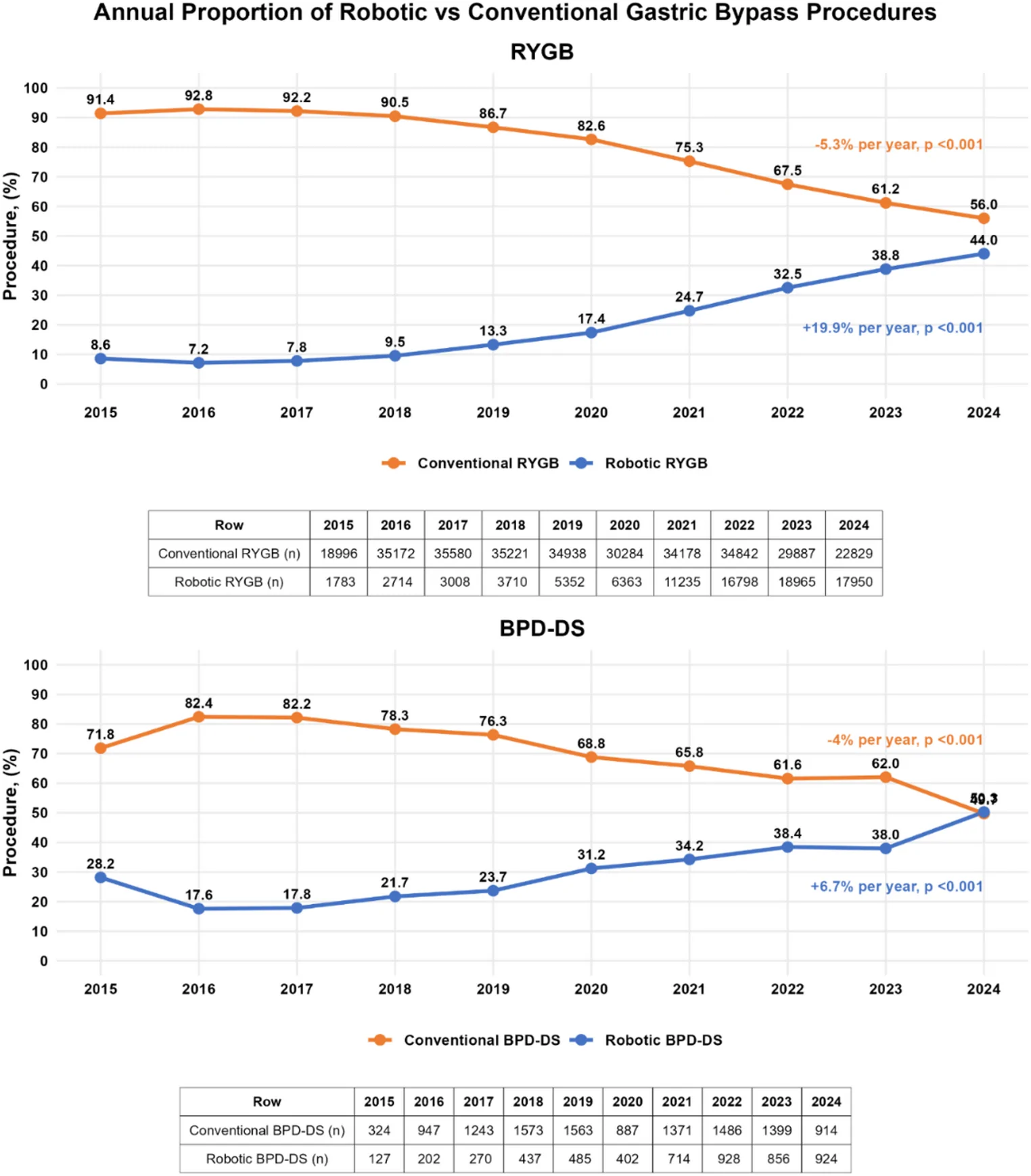
Temporal trends in the annual utilization of robotic versus conventional Roux-en-Y gastric bypass (RYGB) and biliopancreatic diversion with duodenal switch (BPD-DS) procedures from 2015 to 2024. Annual proportions of robotic and laparoscopic approaches are presented separately for RYGB and BPD-DS. Percentages were calculated relative to the total annual procedural volume within each procedure type. Temporal trends in robotic utilization were assessed using weighted linear regression models based on annual procedural counts. Corresponding annual counts for the robotic and laparoscopic approaches are displayed below each panel

Robotic utilization increased progressively over the study period for both procedures, although the magnitude of adoption differed between RYGB and BPD-DS (Fig. 1). Among RYGB procedures, the proportion performed robotically increased from 8.6% in 2015 to 44.0% in 2024, corresponding to an average relative annual increase of 19.9% (*p* < 0.001). Accordingly, the proportion of L-RYGB decreased over the same period.

Among BPD-DS procedures, robotic utilization increased from 28.2% in 2015 to 50.3% in 2024, corresponding to an average relative annual increase of 6.7% (*p* < 0.001). Thus, robotic adoption increased for both RYGB and BPD-DS. Still, the adoption pattern was procedure-specific, with R-RYGB showing a larger relative increase from a lower baseline proportion. In contrast, R-BPD-DS began from a higher baseline robotic proportion and increased more gradually.

### Covariate balance before and after stabilized inverse probability of treatment weighting

**Fig. 2:**
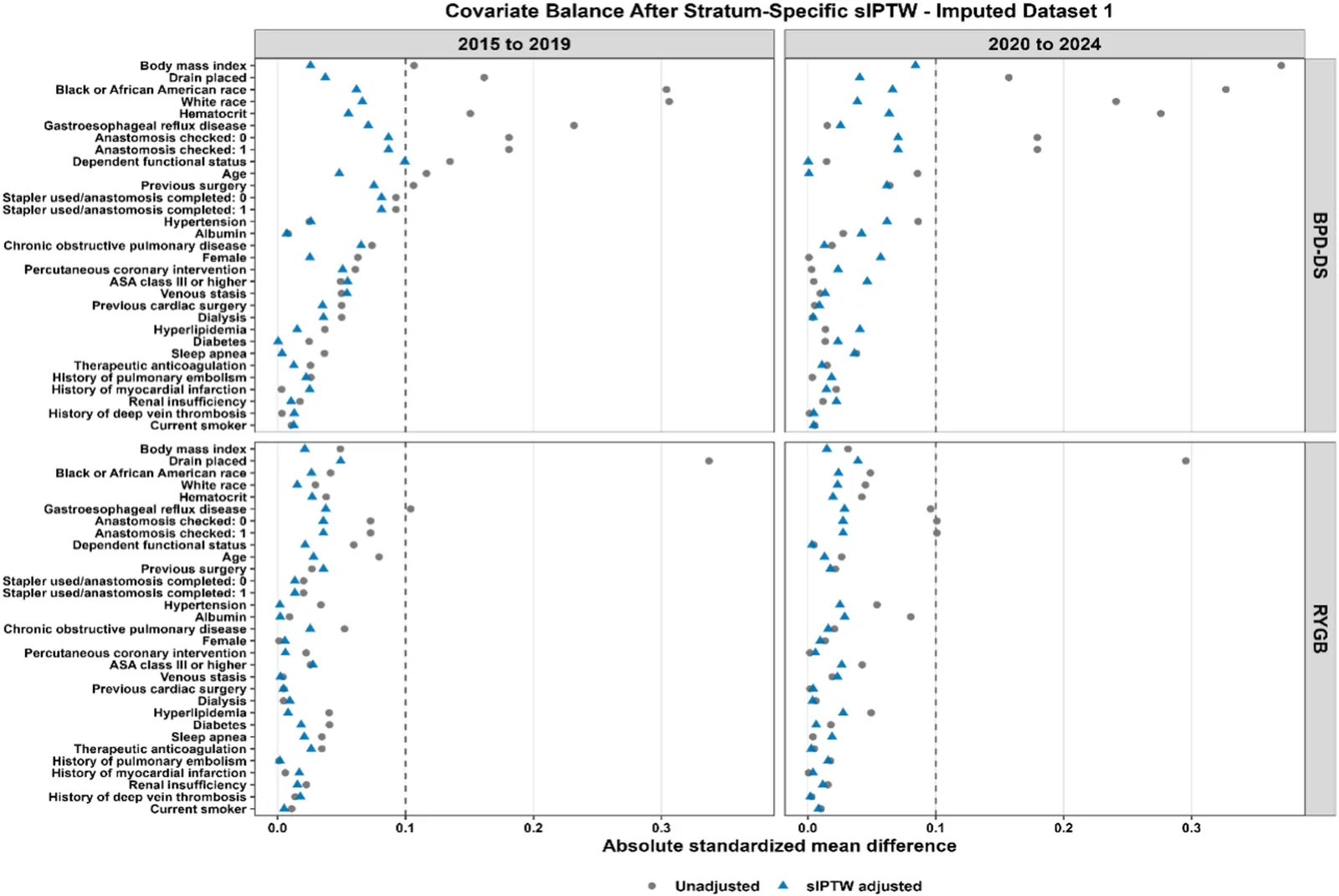
Covariate balance before and after stratum-specific stabilized inverse probability of treatment weighting (sIPTW) in the first imputed dataset. The love plot displays absolute standardized mean differences (aSMDs) for baseline and procedural covariates comparing robotic and laparoscopic approaches within each procedure-period stratum: RYGB 2015–2019, RYGB 2020–2024, BPD-DS 2015–2019, and BPD-DS 2020–2024. Propensity-score models were fitted separately within each procedure-period stratum. Gray circles indicate unadjusted balance and blue triangles indicate balance after sIPTW. The vertical dashed line indicates the predefined threshold for acceptable balance (aSMD < 0.10). After weighting, all covariates demonstrated adequate balance within each procedure-period stratum

Graphical assessment of covariate balance before and after weighting in the first imputed dataset is presented in Fig. 2, whereas the corresponding stratum-specific balance metrics are summarized in Table 1. Propensity-score models were fitted separately within each procedure-period stratum. Before weighting, the magnitude of imbalance varied across strata, with the largest differences observed for drain placement in RYGB during 2015–2019 and 2020–2024 (aSMD = 0.34 and 0.30, respectively), and for body mass index, Black or African American race, hematocrit, White race, and gastroesophageal reflux disease in selected BPD-DS strata. After stratum-specific stabilized IPTW, covariate balance improved substantially across procedure-period strata, with all post-weighting aSMDs below the prespecified threshold for acceptable balance (aSMD < 0.10) in the first imputed dataset. Similar balance patterns were observed across the remaining imputed datasets (Supplementary Figures S2-S6) and in the complete-case sensitivity analysis, in which all post-weighting aSMDs were also < 0.10 (Supplementary Table S4).

**Table 1:** Covariate balance before and after stratum-specific stabilized inverse probability of treatment weighting in the first imputed dataset

Covariate	RYGB 2015–2019		RYGB 2020–2024		BPD-DS 2015–2019		BPD-DS 2020–2024	
	Unadjusted aSMD	sIPTW aSMD	Unadjusted aSMD	sIPTW aSMD	Unadjusted aSMD	sIPTW aSMD	Unadjusted aSMD	sIPTW aSMD
Age	0.08	0.03	0.03	0.01	0.12	0.05	0.09	0.00
Female	0.00	0.01	0.01	0.01	0.06	0.03	0.00	0.06
Body mass index	0.05	0.02	0.03	0.01	0.11	0.03	0.37	0.08
White race	0.03	0.02	0.05	0.02	0.31	0.07	0.24	0.04
Black or African American race	0.04	0.03	0.05	0.02	0.30	0.06	0.33	0.07
Dependent functional status	0.06	0.02	0.00	0.00	0.13	0.10	0.01	0.00
ASA class III or higher	0.03	0.03	0.04	0.03	0.05	0.05	0.00	0.05
Albumin	0.01	0.00	0.08	0.03	0.01	0.01	0.03	0.04
Hematocrit	0.04	0.03	0.04	0.02	0.15	0.06	0.28	0.06
Diabetes	0.04	0.02	0.02	0.01	0.02	0.00	0.01	0.02
Hypertension	0.03	0.00	0.05	0.03	0.03	0.03	0.09	0.06
Hyperlipidemia	0.04	0.01	0.05	0.03	0.04	0.02	0.01	0.04
GERD	0.10	0.04	0.10	0.03	0.23	0.07	0.02	0.03
Therapeutic anticoagulation	0.03	0.03	0.01	0.00	0.03	0.01	0.02	0.01
COPD	0.05	0.03	0.02	0.02	0.07	0.07	0.02	0.01
History of pulmonary embolism	0.00	0.00	0.02	0.02	0.03	0.02	0.00	0.02
Sleep apnea	0.03	0.02	0.00	0.02	0.04	0.00	0.04	0.04
History of myocardial infarction	0.01	0.02	0.00	0.00	0.00	0.03	0.02	0.01
PCI	0.02	0.01	0.00	0.01	0.06	0.05	0.00	0.02
Previous cardiac surgery	0.00	0.00	0.00	0.00	0.05	0.04	0.01	0.01
History of deep vein thrombosis	0.01	0.02	0.00	0.00	0.00	0.01	0.00	0.00
Venous stasis	0.00	0.00	0.02	0.02	0.05	0.05	0.01	0.01
Dialysis	0.00	0.01	0.01	0.00	0.05	0.04	0.00	0.00
Renal insufficiency	0.02	0.02	0.02	0.01	0.02	0.01	0.01	0.02
Previous surgery	0.03	0.04	0.02	0.02	0.11	0.08	0.06	0.06
Current smoker	0.01	0.01	0.01	0.01	0.01	0.01	0.01	0.00
Drain placed	0.34	0.05	0.30	0.04	0.16	0.04	0.16	0.04

Values are absolute standardized mean differences before and after stratum-specific stabilized inverse probability of treatment weighting in the first imputed dataset. Propensity-score models were fitted separately within each procedure-period stratum and excluded conversion to open surgery. Values < 0.10 were considered indicative of acceptable covariate balance. Abbreviations: aSMD, absolute standardized mean difference; ASA, American Society of Anesthesiologists; BPD-DS, biliopancreatic diversion with duodenal switch; RYGB, Roux-en-Y gastric bypass; sIPTW, stabilized inverse probability of treatment weighting; GERD, Gastroesophageal reflux disease; COPD, Chronic obstructive pulmonary disease; PCI, Percutaneous coronary intervention

### Stratified propensity-weighted perioperative and 30-day outcomes

**Fig. 3:**
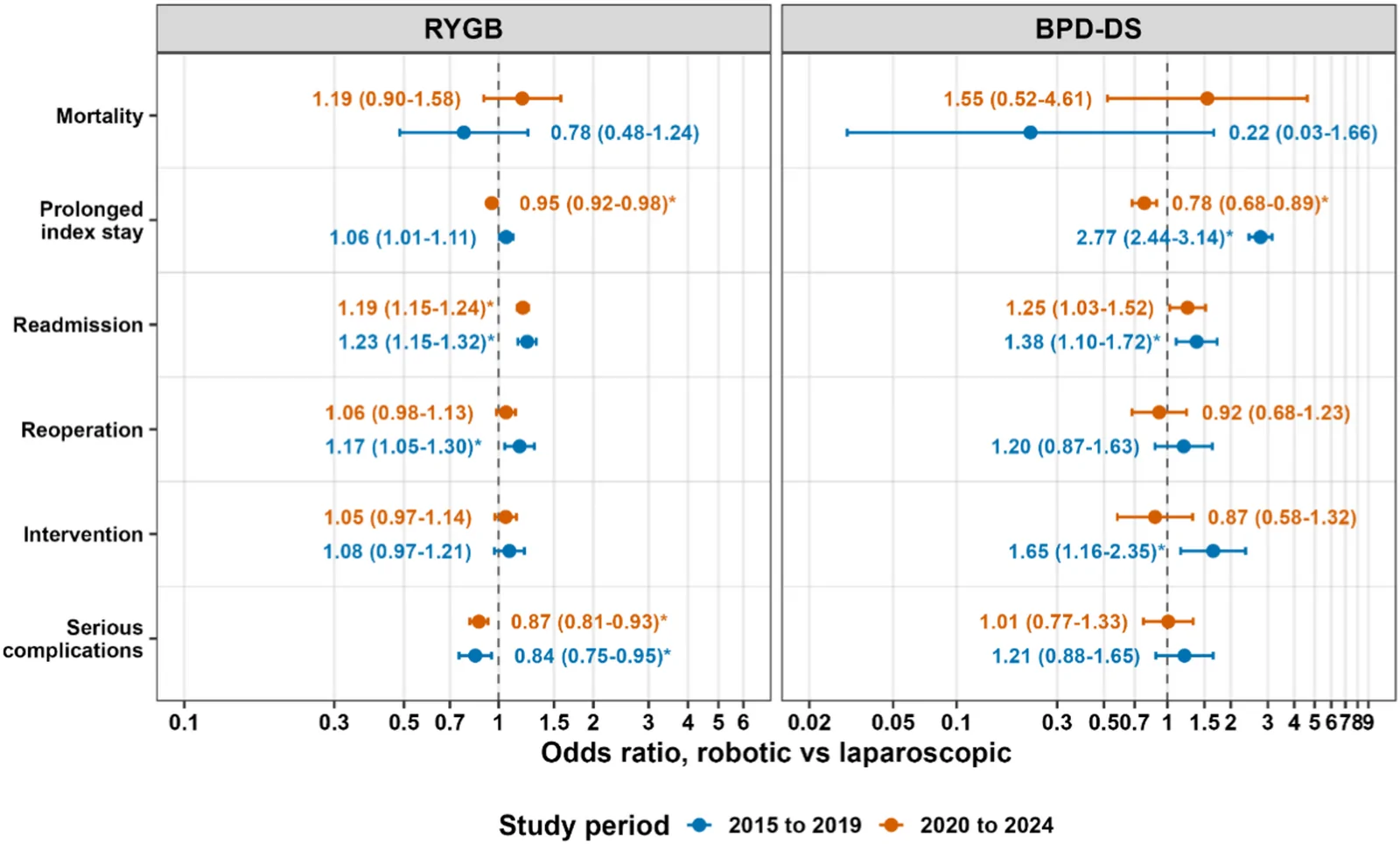
Stratum-specific sIPTW-adjusted associations between robotic and laparoscopic approaches and grouped 30-day endpoints in the pooled multiply imputed analysis. Forest plots present odds ratios (ORs) with 95% confidence intervals (CIs) comparing robotic with laparoscopic approaches within each procedure-period stratum. Propensity-score and outcome models were fitted separately for RYGB 2015–2019, RYGB 2020–2024, BPD-DS 2015–2019, and BPD-DS 2020–2024, and estimates were pooled across the five imputed datasets using Rubin’s rules. ORs > 1 indicate higher odds associated with the robotic approach. Asterisks indicate statistical significance after Benjamini-Hochberg false-discovery-rate adjustment across the full family of 28 procedure-period endpoint comparisons

**Fig. 4:**
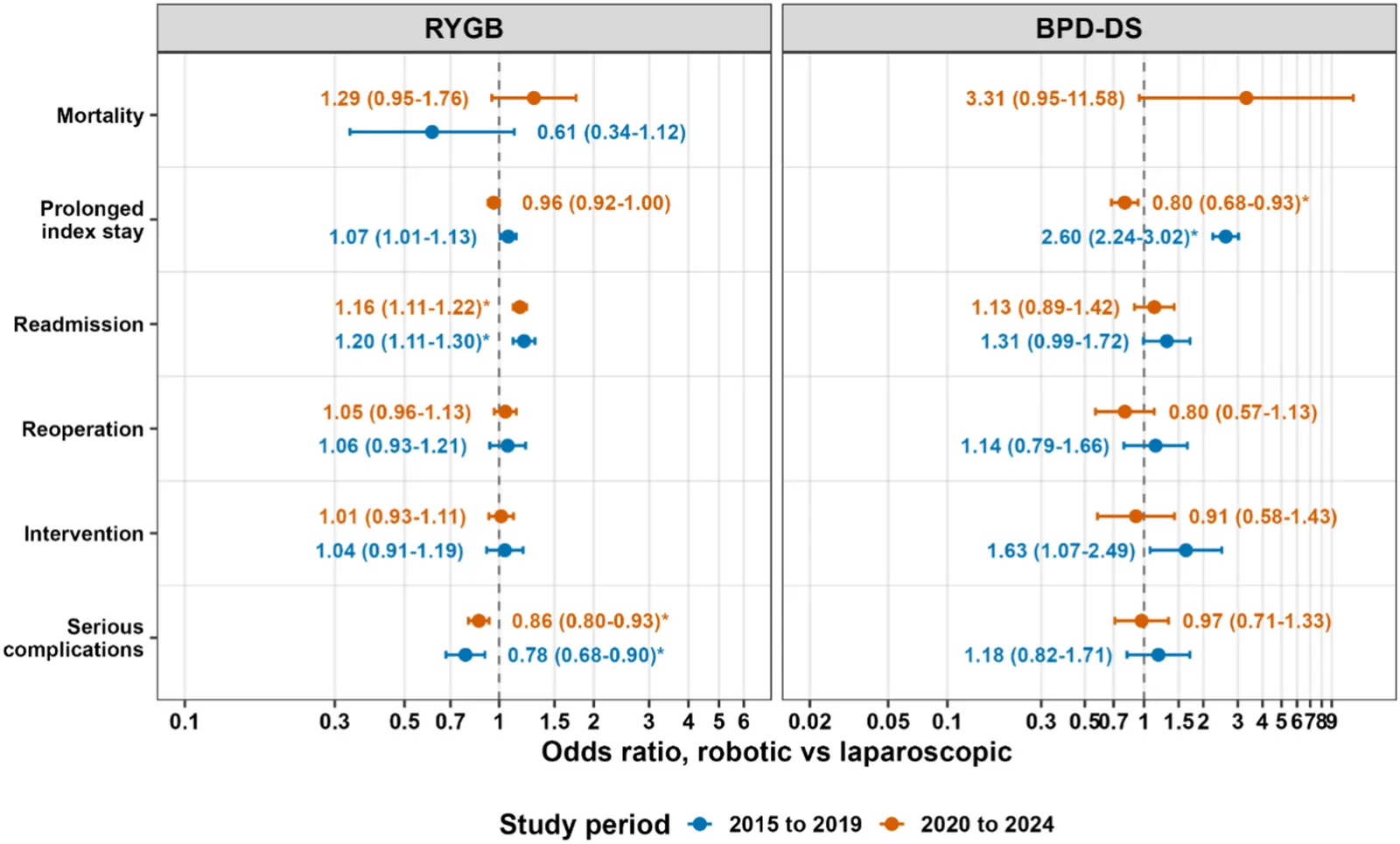
Stratum-specific sIPTW-adjusted associations between robotic and laparoscopic approaches and grouped 30-day endpoints in the complete-case sensitivity analysis. Forest plots present odds ratios (ORs) with 95% confidence intervals (CIs) comparing robotic with laparoscopic approaches within each procedure-period stratum in the complete-case cohort. Propensity-score and outcome models were fitted separately for RYGB 2015–2019, RYGB 2020–2024, BPD-DS 2015–2019, and BPD-DS 2020–2024. ORs > 1 indicate higher odds associated with the robotic approach. Asterisks indicate statistical significance after Benjamini-Hochberg false-discovery-rate adjustment across the full family of 28 procedure-period endpoint comparisons

Perioperative and 30-day outcomes were evaluated separately within each procedure-period stratum using stratum-specific sIPTW (Tables 2 and 3; Fig. 3). Because the revised models were fitted separately within each stratum, no pooled three-way interaction term or corresponding interaction p-value was estimated. Corresponding complete-case results are presented in Supplementary Tables S5 and S6 and Fig. 4. Table 2 provides cell sizes and sIPTW-weighted event rates across the five imputed datasets across the five imputed datasets, allowing interpretation of the denominators underlying each categorical estimate.

**Table 2:** Pooled cell sizes for grouped 30-day endpoints and operative length across procedure-period strata

Outcome	RYGB 2015–2019		RYGB 2020–2024		BPD-DS 2015–2019		BPD-DS 2020–2024	
	Robotic	Laparoscopic	Robotic	Laparoscopic	Robotic	Laparoscopic	Robotic	Laparoscopic
Mortality	20/16,242 (0.1)	219/156,600 (0.1)	80/70,786 (0.1)	140/150,820 (0.1)	1/1,489 (0.1)	25/5,471 (0.5)	8/3,794 (0.2)	7/5,953 (0.1)
Prolonged index stay	2,272/16,242 (15.3)	22,925/156,600 (14.6)	5,056/70,781 (7.5)	11,898/150,810 (7.8)	649/1,489 (40.6)	1,056/5,471 (19.8)	410/3,792 (10.0)	685/5,952 (12.5)
Readmission	1,142/16,242 (7.0)	8,984/156,600 (5.7)	3,813/70,786 (5.4)	6,791/150,820 (4.5)	123/1,489 (8.1)	329/5,471 (6.1)	203/3,794 (5.4)	252/5,953 (4.3)
Reoperation	415/16,242 (2.6)	3,444/156,600 (2.2)	1,227/70,786 (1.7)	2,498/150,820 (1.7)	58/1,489 (3.8)	176/5,471 (3.2)	75/3,794 (2.1)	132/5,953 (2.3)
Intervention	396/16,242 (2.4)	3,464/156,600 (2.2)	984/70,786 (1.4)	1,993/150,820 (1.3)	48/1,489 (3.4)	112/5,471 (2.1)	39/3,794 (1.1)	68/5,953 (1.2)
Serious complications	340/16,242 (2.1)	3,917/156,600 (2.5)	1,302/70,786 (1.8)	3,215/150,820 (2.1)	59/1,489 (3.8)	174/5,471 (3.2)	95/3,794 (2.6)	144/5,953 (2.6)
Operative length	157.63 ± 64.38	117.79 ± 52.67	143.74 ± 57.58	117.57 ± 54.19	214.99 ± 73.33	127.50 ± 67.08	164.22 ± 67.84	123.37 ± 62.33

Values are pooled across the five weighted imputed datasets. Categorical outcomes are presented as events/N with weighted percentages in parentheses; percent signs are omitted from table cells. Operative length is presented as weighted mean ± weighted SD. Prolonged index stay was defined as index hospital stay > = 3 days. Serious complications were defined as a composite of postoperative sepsis, septic shock, organ-space surgical-site infection, deep incisional surgical-site infection, transfusion or bleeding, pulmonary embolism, venous thrombosis requiring therapy, pneumonia, ventilator use, acute renal failure, progressive renal insufficiency, cardiac arrest requiring CPR, myocardial infarction, and stroke. Abbreviations: BPD-DS, biliopancreatic diversion with duodenal switch; RYGB, Roux-en-Y gastric bypass; SD, standard deviation; sIPTW, stabilized inverse probability of treatment weighting

**Table 3:** Stratified propensity-weighted associations between robotic and laparoscopic approaches and grouped 30-day endpoints and operative length

Outcome	RYGB 2015–2019			RYGB 2020–2024			BPD-DS 2015–2019			BPD-DS 2020–2024		
	OR/MD (95% CI)	Unadjusted *p*	BH-adjusted *p*	OR/MD (95% CI)	Unadjusted *p*	BH-adjusted *p*	OR/MD (95% CI)	Unadjusted *p*	BH-adjusted *p*	OR/MD (95% CI)	Unadjusted *p*	BH-adjusted *p*
Mortality	0.78 (0.48–1.24)	0.287	0.335	1.19 (0.90–1.58)	0.229	0.305	0.22 (0.03–1.66)	0.143	0.222	1.55 (0.52–4.61)	0.433	0.485
Prolonged index stay	1.06 (1.01–1.11)	**0.029**	0.051	0.95 (0.92–0.98)	**0.005**	**0.012**	2.77 (2.44–3.14)	**< 0.001**	**0.003**	0.78 (0.68–0.89)	**< 0.001**	**0.003**
Readmission	1.23 (1.15–1.32)	**< 0.001**	**0.003**	1.19 (1.15–1.24)	**< 0.001**	**0.003**	1.38 (1.10–1.72)	**0.005**	**0.012**	1.25 (1.03–1.52)	**0.027**	0.050
Reoperation	1.17 (1.05–1.30)	**0.006**	**0.012**	1.06 (0.98–1.13)	0.133	0.219	1.20 (0.87–1.63)	0.263	0.320	0.92 (0.68–1.23)	0.559	0.580
Intervention	1.08 (0.97–1.21)	0.161	0.237	1.05 (0.97–1.14)	0.200	0.280	1.65 (1.16–2.35)	**0.006**	**0.012**	0.87 (0.58–1.32)	0.521	0.561
Serious complications	0.84 (0.75–0.95)	**0.005**	**0.012**	0.87 (0.81–0.93)	**< 0.001**	**0.003**	1.21 (0.88–1.65)	0.242	0.308	1.01 (0.77–1.33)	0.945	0.945
Operative length	39.83 (38.71–40.96)	**< 0.001**	**0.003**	26.17 (25.64–26.69)	**< 0.001**	**0.003**	87.49 (83.10-91.88)	**< 0.001**	**0.003**	40.85 (38.04–43.66)	**< 0.001**	**0.003**

Values are presented as odds ratios with 95% confidence intervals for binary endpoints and mean differences with 95% confidence intervals for operative length. All estimates compare robotic with laparoscopic approaches within each procedure-period stratum. Propensity-score models were fitted separately within each procedure-period stratum and excluded conversion to open surgery. Prolonged index stay was defined as index hospital stay > = 3 days. Serious complications were defined as a composite of postoperative sepsis, septic shock, organ-space surgical-site infection, deep incisional surgical-site infection, transfusion or bleeding, pulmonary embolism, venous thrombosis requiring therapy, pneumonia, ventilator use, acute renal failure, progressive renal insufficiency, cardiac arrest requiring CPR, myocardial infarction, and stroke. Benjamini-Hochberg adjusted p values were calculated across the full family of 28 procedure-period endpoint comparisons. Abbreviations: BH, Benjamini-Hochberg; BPD-DS, biliopancreatic diversion with duodenal switch; CI, confidence interval; MD, mean difference; OR, odds ratio; RYGB, Roux-en-Y gastric bypass

Among RYGB procedures, robotic surgery was associated with higher odds of readmission in both periods (2015–2019: OR 1.23 [95% CI, 1.15–1.32]; 2020–2024: OR 1.19 [95% CI, 1.15–1.24]), with both associations remaining significant after Benjamini-Hochberg correction. Reoperation was higher with robotic RYGB during 2015–2019 (OR 1.17 [95% CI, 1.05–1.30]), whereas the 2020–2024 estimate was not statistically significant. Serious complications were less frequent with robotic RYGB in both periods (2015–2019: OR 0.84 [95% CI, 0.75–0.95]; 2020–2024: OR 0.87 [95% CI, 0.81–0.93]). Prolonged index stay showed a small increase with robotic RYGB during 2015–2019 that did not remain significant after multiplicity adjustment (OR 1.06 [95% CI, 1.01–1.11]; BH-adjusted *p* = 0.051), and a small reduction during 2020–2024 (OR 0.95 [95% CI, 0.92–0.98]). Mortality and intervention were not significantly different between approaches in either RYGB period.

Among BPD-DS procedures, robotic surgery was associated with higher odds of prolonged index stay during 2015–2019 (OR 2.77 [95% CI, 2.44–3.14]) but lower odds during 2020–2024 (OR 0.78 [95% CI, 0.68–0.89]). Readmission was higher with robotic BPD-DS in both periods (2015–2019: OR 1.38 [95% CI, 1.10–1.72]; 2020–2024: OR 1.25 [95% CI, 1.03–1.52]), with the 2020–2024 association at the threshold of significance after multiplicity adjustment. Intervention was higher with robotic BPD-DS during 2015–2019 (OR 1.65 [95% CI, 1.16–2.35]) but not during 2020–2024. Mortality, reoperation, and serious complications did not differ significantly between approaches in either BPD-DS period. Interpretation of BPD-DS estimates, particularly mortality and early-era outcomes, should consider the smaller cell sizes shown in Table 2.

Robotic surgery was associated with substantially longer operative length across both procedures and periods. The adjusted mean difference was 39.83 min for RYGB during 2015–2019, 26.17 min for RYGB during 2020–2024, 87.49 min for BPD-DS during 2015–2019, and 40.85 min for BPD-DS during 2020–2024, all remaining significant after Benjamini-Hochberg correction. Findings from the complete-case sensitivity analysis were generally consistent with the multiply imputed analysis, including persistently longer operative length with robotic procedures and similar directional patterns for grouped 30-day endpoints (Supplementary Tables S5 and S6; Fig. 4).

## Discussion

In this decade-long MBSAQIP analysis, robotic and laparoscopic approaches were compared within two distinct anastomotic MBS procedures: Roux-en-Y gastric bypass (RYGB) and biliopancreatic diversion with duodenal switch (BPD-DS). Robotic utilization increased for both operations, although the pace of adoption differed descriptively between RYGB and BPD-DS. Across the prespecified procedure-period strata, the most consistent finding was a substantially longer operative duration with the robotic approach, with a smaller magnitude in the later period. Higher odds of 30-day readmission were also observed with robotic surgery in both RYGB and BPD-DS. Additional associations varied across the procedure-period strata; however, because the revised analysis was based on prespecified stratified comparisons rather than formal interaction testing, these between-stratum differences should not be interpreted as statistical evidence of effect modification. In RYGB, the robotic approach was associated with lower odds of the serious-complication composite in both periods and higher readmission in both periods; reoperation was higher only during 2015–2019, while mortality and unplanned intervention did not differ significantly between approaches. In BPD-DS, robotic surgery was associated in the primary analysis with higher readmission in both periods, higher unplanned intervention during 2015–2019, and opposing associations with prolonged index stay across the two periods, whereas mortality, reoperation, and serious complications did not differ significantly between approaches. Selected BPD-DS associations, particularly readmission and early-period intervention, were attenuated in the complete-case sensitivity analysis and should therefore be interpreted cautiously.

### Robotic adoption trends

The increase in robotic utilization observed in the present study is consistent with the broader expansion of robotic MBS across national MBSAQIP data. Earlier national analyses reported rising robotic use across MBS procedures, from 6.7% in 2015 to 10.3% in 2018 and from 6.2% in 2015 to 13.5% in 2019 [14, 15]. Analyses extending to 2020 similarly demonstrated continued growth, with robotic RYGB increasing from 6.8% to 16.7% between 2015 and 2020 [16], while robotic MBS overall increased 1.96-fold between 2015 and 2018 [17]. These earlier estimates are consistent with the early-period values in the present cohort and demonstrate that the expansion of robotic MBS continued through 2024.

Importantly, adoption was procedure-specific. R-RYGB increased steeply from a relatively low baseline, rising from 8.6% in 2015 to 44.0% in 2024, whereas R-BPD-DS began from a substantially higher baseline of 28.2% and increased more gradually to 50.3%. These differing trajectories may partly reflect differences in procedural complexity and the context in which robotic technology is adopted. BPD-DS is technically complex and has historically not achieved widespread acceptance among patients or bariatric surgeons; robotic assistance has been proposed to facilitate technically demanding components through improved freedom of movement, precision, and maneuverability, including performance of the duodenoileal anastomosis [18]. The contrasting utilization trajectories therefore suggest different patterns of robotic adoption between RYGB and BPD-DS, although the MBSAQIP data cannot determine the surgeon-, center-, or practice-level mechanisms underlying these differences.

### R-RYGB versus L-RYGB

Within RYGB, the most reproducible difference was a longer operative duration with R-RYGB, present in both study periods, with the adjusted mean difference smaller in 2020–2024 than in 2015–2019 (39.83 versus 26.17 min). This magnitude aligns closely with prior national estimates of an additional 26 to 38 min for robotic MBS [15] and with propensity-matched MBSAQIP data through 2023 showing that robotic operative times remained roughly 18% longer for RYGB in every year despite a ten-fold rise in robotic case volume, and a substantial increase in its learning curve [19]. The persistence of this operative-time difference despite increasing robotic utilization suggests that factors beyond the early learning curve may contribute, although the present analysis cannot determine the mechanism. In parallel, R-RYGB was associated with higher odds of 30-day readmission in both periods, a signal echoed across contemporary evidence: a national MBSAQIP analysis reported higher adjusted 30-day readmission and intervention rates with robotic MBS [15], a meta-analysis found a modestly higher reoperation rate without a reduction in overall complications [11], and a large propensity-matched series linked longer robotic operative times to worse 30-day outcomes, including higher reoperation, readmission, and reintervention [19]. For RYGB, therefore, the robotic approach has not translated its technical attributes into a short-term reduction in unplanned postoperative events.

The short-term safety profile was nonetheless mixed. R-RYGB was associated with lower odds of the serious-complication composite in both periods (2015–2019: OR 0.84, 95% CI 0.75–0.95; 2020–2024: OR 0.87, 95% CI 0.81–0.93), while 30-day readmission remained higher with the robotic approach in both strata. Reoperation was higher with R-RYGB during 2015–2019 but not during 2020–2024. Prolonged index stay showed only a small increase during 2015–2019 that did not remain significant after multiplicity adjustment, whereas a small reduction was observed during 2020–2024; mortality and unplanned intervention did not differ significantly between approaches in either period. These findings are broadly consistent with previous registry and meta-analytic evidence suggesting comparable mortality and no consistent reduction in overall postoperative morbidity with robotic compared with laparoscopic bariatric surgery [7, 11]. Importantly, the complete-case sensitivity analysis reinforced the principal RYGB findings, with persistently higher readmission, lower odds of serious complications, and longer operative duration, whereas the early-period reoperation and prolonged-stay associations were attenuated. Taken together, the findings suggest a mixed short-term outcome profile rather than a uniform safety advantage for either approach: the robotic approach was associated with fewer serious complications but more readmissions and a persistent operative-time burden. This interpretation is consistent with the broader literature indicating that robotic RYGB has not demonstrated a consistent overall perioperative benefit sufficient to offset its longer operative duration [11, 19].

### R-BPD-DS versus L-BPD-DS

Similarly, R-BPD-DS was associated with a longer operative duration than L-BPD-DS, with its magnitude exceeding that observed in RYGB (87.49 min in 2015–2019 and 40.85 min in 2020–2024). This might be due to the greater technical demands of BPD-DS, a multi-quadrant operation combining SG with duodeno-ileal and ileo-ileal anastomoses, and to its status as the least commonly performed primary MBS procedure, attributed in part to its longer operative duration and higher technical complication rates [20]. A previous MBSAQIP comparison of R-BPD-DS and L-BPD-DS similarly reported longer operative duration with the robotic approach and a one-day longer median hospital stay in 2015, with comparable length of stay between approaches in subsequent years [21]. The smaller operative-time difference observed in the later period is compatible with a learning-curve effect, although the present analysis did not formally test a temporal interaction: a dedicated analysis identified a learning curve of approximately 50 cases for R-BPD-DS, after which operative duration shortened and complications declined, with surgeon case number strongly associated with outcomes [20].

Beyond the persistent operative-time difference, R-BPD-DS was associated with higher odds of 30-day readmission in both procedure-period strata and with higher odds of unplanned intervention during 2015–2019. Prolonged index stay was higher with the robotic approach during 2015–2019 but lower during 2020–2024, whereas mortality, reoperation, and serious complications did not differ significantly between approaches in either period. In the complete-case sensitivity analysis, however, the BPD-DS readmission associations were attenuated and no longer statistically significant after multiplicity adjustment, while the early-period intervention estimate remained directionally elevated but narrowly missed the adjusted significance threshold. These findings further support cautious interpretation of the BPD-DS associations. Importantly, the revised analysis used separate propensity-score models within each BPD-DS procedure-period stratum, and all measured covariates achieved acceptable post-weighting balance (aSMD < 0.10). Therefore, the remaining uncertainty surrounding the BPD-DS estimates primarily reflects smaller sample sizes, lower event counts, and wider confidence intervals, rather than inadequate demonstration of within-procedure covariate balance.

These findings should consequently be interpreted with caution and considered hypothesis-generating. They should also be considered alongside a largely reassuring current literature: the national duodenal switch comparison found no significant differences in organ-space infection or sepsis and concluded that most short-term outcomes were comparable between robotic and laparoscopic approaches [21]; a broader MBSAQIP analysis similarly found no significant between-approach differences in major 30-day outcomes for duodenal switch [7]; and a single-center robotic BPD-DS series reported low early morbidity, a 5% 30-day readmission rate, and no anastomotic leak or mortality [22]. A possible explanation for some of the early-period signals observed here is accumulating experience during robotic adoption rather than a hazard intrinsic to the platform; national MBSAQIP data have shown declining readmission and reintervention rates over time across robotic MBS procedures overall [17]. To our knowledge, this is among the largest procedure-specific, weighted comparisons of R-BPD-DS and L-BPD-DS reported to date; nonetheless, given the limited volume and the exploratory nature of the outcome signals, these findings should be interpreted with caution and treated as hypothesis-generating, pending prospective, adequately powered, procedure-specific evaluation.

### Comparison with previous literature

This study observed an overall pattern consistent with current evidence on robotic MBS, in which the robotic approach is repeatedly associated with longer operative times and higher costs, while short-term complication rates and mortality are generally comparable with laparoscopy [7, 11, 22, 23]. Much of the literature supporting the “comparable outcomes” conclusion, however, derives from analyses that combined anatomically different MBS operations, examined shorter or earlier time windows, or were underpowered for procedure- and era-specific contrasts, and contemporary randomized evidence remains scarce. For RYGB, randomized evidence is limited to a small single-center comparison conducted during an early phase of robotic adoption [24, 25], while randomized comparative evidence for robotic versus laparoscopic BPD-DS remains absent [25]. By comparing approaches within each operation across a full decade using prespecified procedure-period strata, the present analysis provides granularity that pooled or short-window studies cannot. It identifies a readmission signal observed across both operations alongside additional stratum-specific associations that may be obscured within a combined ‘gastric bypass’ or all-procedure comparison. These between-stratum patterns are descriptive and should not be interpreted as formal evidence of effect modification. These findings are best interpreted as refining rather than contradicting the existing evidence, since the absence of a mortality difference and the persistent operative-time disadvantage are preserved, while the additional associations are observational and, for BPD-DS, exploratory. Taken together with evidence that robotic MBS is associated with higher procedural costs without a consistent overall perioperative benefit [11, 23], the persistent operative-time gap documented here reinforces the case for randomized, procedure-specific, and cost-inclusive evaluation of the robotic platform in distinct MBS operations.

### Strengths and limitations

This analysis has several strengths. It draws on a decade of national MBSAQIP data (2015–2024), providing, to our knowledge, among the longest and largest cohorts comparing robotic and laparoscopic approaches within RYGB and within BPD-DS. The design was procedure-specific rather than pooling anatomically distinct operations; confounding was addressed within a target-trial framework using stratum-specific stabilized inverse probability of treatment weighting from generalized boosted models, with covariate balance demonstrated separately within each procedure-period stratum; multiple imputation and a complete-case sensitivity analysis were performed; multiplicity was addressed using Benjamini-Hochberg false-discovery-rate adjustment; and reporting followed the RECORD guideline. The inclusion of BPD-DS provides one of the largest weighted comparisons of this infrequently studied operation to date.

Several limitations should be acknowledged. First, as an observational analysis, weighting could balance only measured covariates, and residual confounding remains possible. MBSAQIP does not capture surgeon or institutional robotic volume or learning-curve stage, which may influence approach selection, operative time, and outcomes [26]. Therefore, attenuation of some associations in the later period cannot be attributed specifically to increasing experience.

Second, although propensity-score models were fitted separately within each procedure-period stratum and achieved acceptable measured covariate balance, the BPD-DS strata remained substantially smaller than the corresponding RYGB strata. Consequently, BPD-DS estimates were less precise, particularly for infrequent outcomes, and should be interpreted cautiously.

Third, MBSAQIP operative-approach and procedure-type coding changed during the study period. Although year-specific harmonization algorithms were applied, residual misclassification remains possible. The 2015–2019 and 2020–2024 periods were prespecified to reflect these coding eras and should not be interpreted as a statistically identified temporal breakpoint. The analysis was not designed to determine the exact year in which approach–outcome associations changed.

Fourth, registry coding does not distinguish fully robotic from hybrid procedures [16]. Fifth, outcomes were limited to 30 days and excluded weight loss, metabolic, nutritional, long-term, and cost outcomes [8]. Sixth, although Benjamini-Hochberg false-discovery-rate adjustment was applied across the modeled comparisons, the number of evaluated endpoints and the sparse event counts for selected BPD-DS outcomes warrant cautious interpretation.

### Future directions

Prospective, procedure-specific studies that incorporate surgeon and institutional robotic volume and learning-curve stage are needed to determine whether the associations observed here persist once operative experience is accounted for, and randomized comparisons remain absent for BPD-DS. Given the consistent operative-time penalty, formal cost-effectiveness evaluation is warranted, alongside longer-term outcomes, weight loss, metabolic and nutritional status, and durability, that fall outside the 30-day window yet are central to the value of each operation, particularly BPD-DS. The exploratory signals identified for robotic BPD-DS specifically merit dedicated, adequately powered evaluation.

## Conclusion

In this decade-long national analysis, robotic utilization increased substantially for both RYGB and BPD-DS. Across the prespecified procedure-period strata, the robotic approach was consistently associated with longer operative duration, while higher 30-day readmission was observed in both procedures. Additional associations varied across individual procedure-period strata and should be interpreted as stratum-specific findings rather than evidence of formal effect modification. R-RYGB demonstrated a mixed short-term outcome profile, with lower odds of serious complications but higher readmission and a persistent operative-time burden. BPD-DS estimates were less precise, and selected associations attenuated in the complete-case analysis, supporting cautious and hypothesis-generating interpretation. Prospective, procedure-specific studies incorporating operative experience, longer-term outcomes, and cost are needed to define the clinical value of continued robotic adoption.

## Supplementary Information

Below is the link to the electronic supplementary material.

Supplementary Material 1

## Data Availability

The data analyzed in this study were obtained from the Metabolic and Bariatric Surgery Accreditation and Quality Improvement Program Participant Use File program. Access is subject to the program’s application, eligibility, and data-use requirements. Under the applicable data-use agreement, the authors are not permitted to publicly redistribute the patient-level data.

## Code availability

The analytic code may be made available by the corresponding author upon reasonable request, subject to the restrictions of the MBSAQIP data-use agreement.
