# Supplementary material for: Procedure-specific outcomes of robotic versus laparoscopic Roux-en-Y gastric bypass and biliopancreatic diversion with duodenal switch across a decade of MBSAQIP adoption
Source: J Robot Surg. 2026 Sep 4;20(1):953. doi: 10.1007/s11701-026-03865-w (PMC13545063; doi:10.1007/s11701-026-03865-w)
Supplement: Supplementary file 1 [file 11701_2026_3865_MOESM1_ESM.docx]

| **Table S1:** Proportion of missing data for baseline and procedural variables by surgical approach | | | |
| --- | --- | --- | --- |
| **Variables** | **Robotic Approach** | **Laparoscopic Approach** | **Max missing** |
| Albumin | 24.17 | 18.59 | 24.17 |
| Race | 10.71 | 7.65 | 10.71 |
| Hematocrit | 9.21 | 8.39 | 9.21 |
| Anastomosis checked | 0.87 | 0.50 | 0.87 |
| Body mass index | 0.32 | 0.17 | 0.32 |
| Functional status | 0.07 | 0.11 | 0.11 |
| ASA class | 0.10 | 0.04 | 0.10 |
| Sex | 0.03 | 0.05 | 0.05 |
| Age | 0.03 | 0.03 | 0.03 |
| Bleeding units | 0.001 | 0.000 | 0.001 |

Values are presented as percentages of missing observations for each variable within the robotic and laparoscopic procedure groups. “Max missing” represents the highest proportion of missingness observed across the two groups for each variable. SG, sleeve gastrectomy; ASA, American Society of Anesthesiologists.

| **Table S2:** Assessment of associations between covariates and missingness for albumin, hematocrit, and race | | | | | | | |
| --- | --- | --- | --- | --- | --- | --- | --- |
|  |  | **Albumin** | | **HCT** | | **Race** | |
| **predictor** | **test** | **p** | **associated with missingness** | **p** | **associated with missingness** | **p** | **associated with missingness** |
| Year | Chi-square | <0.001 | Yes | <0.001 | Yes | <0.001 | Yes |
| ASA class | Chi-square |  | No |  | No |  | No |
| Length of stay | Wilcoxon rank-sum | <0.001 | Yes | 0.056 | No | <0.001 | Yes |
| Procedure type | Chi-square |  | No |  | No |  | No |
| Body mass index | Wilcoxon rank-sum | 0.028 | Yes | 0.003 | Yes | <0.001 | Yes |
| Hyperlipidemia | Chi-square | <0.001 | Yes | 0.101 | No | <0.001 | Yes |
| Current smoker | Chi-square | <0.001 | Yes | <0.001 | Yes | <0.001 | Yes |
| Functional status | Chi-square | <0.001 | Yes | 0.002 | Yes | <0.001 | Yes |
| Therapeutic anticoagulation | Chi-square | <0.001 | Yes | 0.070 | No | <0.001 | Yes |
| Venous stasis | Chi-square | <0.001 | Yes | <0.001 | Yes | <0.001 | Yes |
| Anastomosis checked | Chi-square | 0.433 | No | 0.089 | No | <0.001 | Yes |
| Gastroesophageal reflux disease | Chi-square | <0.001 | Yes | <0.001 | Yes | <0.001 | Yes |
| Diabetes | Chi-square | <0.001 | Yes | 0.013 | Yes | <0.001 | Yes |
| History of deep vein thrombosis | Chi-square | 0.007 | Yes | <0.001 | Yes | <0.001 | Yes |
| Drain placed | Chi-square | <0.001 | Yes | <0.001 | Yes | <0.001 | Yes |
| History of pulmonary embolism | Chi-square | <0.001 | Yes | 0.019 | Yes | <0.001 | Yes |
| Chronic obstructive pulmonary disease | Chi-square | 0.001 | Yes | <0.001 | Yes | <0.001 | Yes |
| Sex | Chi-square |  | No |  | No |  | No |
| Age | Wilcoxon rank-sum | <0.001 | Yes | 0.166 | No | <0.001 | Yes |
| Renal insufficiency | Chi-square | 0.001 | Yes | 0.301 | No | <0.001 | Yes |
| Previous cardiac surgery | Chi-square | 0.198 | No | 0.307 | No | <0.001 | Yes |
| Dialysis | Chi-square | 0.360 | No | <0.001 | Yes | 0.081 | No |
| Reoperation within 30 days | Chi-square | 0.100 | No | 1.000 | No | <0.001 | Yes |
| History of myocardial infarction | Chi-square | 0.003 | Yes | 0.004 | Yes | <0.001 | Yes |
| Sleep apnea | Chi-square | 0.023 | Yes | 0.124 | No | <0.001 | Yes |
| Progressive renal insufficiency | Chi-square | 0.031 | Yes | 0.008 | Yes | <0.001 | Yes |
| Bleeding units | Wilcoxon rank-sum | 0.031 | Yes | 0.129 | No | 0.714 | No |
| Converted to open | Chi-square | 0.217 | No | 1.000 | No | <0.001 | Yes |
| Organ/space surgical site infection | Chi-square | 0.009 | Yes | 0.191 | No | <0.001 | Yes |
| Deep incisional surgical site infection | Chi-square | 0.608 | No | 0.137 | No | 0.253 | No |
| Percutaneous coronary intervention | Chi-square | 0.002 | Yes | <0.001 | Yes | <0.001 | Yes |
| Intervention within 30 days | Chi-square | 0.118 | No | 0.629 | No | <0.001 | Yes |
| Postoperative ventilator requirement | Chi-square | 0.279 | No | 0.649 | No | 0.034 | Yes |
| Superficial incisional surgical site infection | Chi-square | 0.001 | Yes | 0.388 | No | <0.001 | Yes |
| Septic shock | Chi-square | 0.692 | No | 0.749 | No | 0.006 | Yes |
| Readmission within 30 days | Chi-square | 0.648 | No | 0.077 | No | <0.001 | Yes |
| Hypertension | Chi-square | <0.001 | Yes | <0.001 | Yes | <0.001 | Yes |
| Sepsis | Chi-square | 0.274 | No | 0.339 | No | 0.002 | Yes |
| Pneumonia | Chi-square | 0.550 | No | 0.270 | No | <0.001 | Yes |
| Death within 30 days | Chi-square | 0.219 | No | 0.253 | No | <0.001 | Yes |
| Previous surgery | Chi-square | 0.158 | No | 1.000 | No | <0.001 | Yes |
| Operative length | Wilcoxon rank-sum | <0.001 | Yes | <0.001 | Yes | <0.001 | Yes |
| STAPLING_PROC | Chi-square | 0.020 | Yes | 0.659 | No | 0.004 | Yes |
| **Total number of associated predictors** | | **26** | | **17** | | **37** | |

Associations between each predictor and missingness in albumin, hematocrit (HCT), and race were assessed using chi-square tests for categorical variables and Wilcoxon rank-sum tests for continuous variables. P values indicate the statistical significance of the association between each predictor and missingness for the respective variable. “Associated with missingness” denotes whether the predictor was significantly associated with missingness at a threshold of p < 0.05. The total number of associated predictors represents the count of variables significantly associated with missingness for each outcome.

**Figure S1:** Convergence diagnostics for multiple imputation of albumin, hematocrit, and race across iterations. The figure displays trace plots of the mean and standard deviation for imputed variables (albumin, hematocrit [HCT], and race harmonized) across iterations of the multiple imputation process. Separate lines represent individual imputed datasets. Stability of the chains across iterations indicates adequate convergence of the imputation models.

**Table S3:** Distribution and diagnostics of stabilized inverse probability of treatment weights across imputed datasets using stratum-specific propensity-score models

| **Imputation** | **Procedure-period stratum** | **Surgery group** | **n** | **Effective n** | **Mean weight** | **p99 weight** | **Max weight** |
| --- | --- | --- | --- | --- | --- | --- | --- |
| 1 | RYGB 2015-2019 | Laparoscopic approach | 156,600 | 156472.17 | 1.00 | 1.05 | 1.17 |
| 1 | RYGB 2015-2019 | Robotic approach | 16,242 | 14666.84 | 0.98 | 2.04 | 2.30 |
| 1 | RYGB 2020-2024 | Laparoscopic approach | 150,820 | 149694.87 | 1.00 | 1.13 | 1.18 |
| 1 | RYGB 2020-2024 | Robotic approach | 70,786 | 67026.16 | 0.99 | 2.02 | 2.31 |
| 1 | BPD-DS 2015-2019 | Laparoscopic approach | 5,471 | 5429.38 | 1.00 | 1.30 | 1.44 |
| 1 | BPD-DS 2015-2019 | Robotic approach | 1,489 | 1372.28 | 0.95 | 1.64 | 2.39 |
| 1 | BPD-DS 2020-2024 | Laparoscopic approach | 5,953 | 5746.85 | 0.99 | 1.67 | 1.84 |
| 1 | BPD-DS 2020-2024 | Robotic approach | 3,794 | 3505.36 | 0.97 | 1.83 | 2.50 |
| 2 | RYGB 2015-2019 | Laparoscopic approach | 156,600 | 156472.49 | 1.00 | 1.05 | 1.15 |
| 2 | RYGB 2015-2019 | Robotic approach | 16,242 | 14668.56 | 0.98 | 2.05 | 2.27 |
| 2 | RYGB 2020-2024 | Laparoscopic approach | 150,820 | 149694.13 | 1.00 | 1.13 | 1.18 |
| 2 | RYGB 2020-2024 | Robotic approach | 70,786 | 67021.89 | 0.99 | 2.02 | 2.37 |
| 2 | BPD-DS 2015-2019 | Laparoscopic approach | 5,471 | 5430.66 | 1.00 | 1.27 | 1.44 |
| 2 | BPD-DS 2015-2019 | Robotic approach | 1,489 | 1371.76 | 0.95 | 1.63 | 2.54 |
| 2 | BPD-DS 2020-2024 | Laparoscopic approach | 5,953 | 5740.37 | 0.99 | 1.70 | 1.84 |
| 2 | BPD-DS 2020-2024 | Robotic approach | 3,794 | 3502.81 | 0.97 | 1.86 | 2.27 |
| 3 | RYGB 2015-2019 | Laparoscopic approach | 156,600 | 156472.99 | 1.00 | 1.05 | 1.15 |
| 3 | RYGB 2015-2019 | Robotic approach | 16,242 | 14675.39 | 0.98 | 2.05 | 2.28 |
| 3 | RYGB 2020-2024 | Laparoscopic approach | 150,820 | 149700.56 | 1.00 | 1.13 | 1.18 |
| 3 | RYGB 2020-2024 | Robotic approach | 70,786 | 67033.06 | 0.99 | 2.02 | 2.35 |
| 3 | BPD-DS 2015-2019 | Laparoscopic approach | 5,471 | 5431.63 | 1.00 | 1.27 | 1.43 |
| 3 | BPD-DS 2015-2019 | Robotic approach | 1,489 | 1379.08 | 0.94 | 1.63 | 2.40 |
| 3 | BPD-DS 2020-2024 | Laparoscopic approach | 5,953 | 5750.87 | 0.99 | 1.66 | 1.79 |
| 3 | BPD-DS 2020-2024 | Robotic approach | 3,794 | 3508.77 | 0.97 | 1.81 | 2.28 |
| 4 | RYGB 2015-2019 | Laparoscopic approach | 156,600 | 156472.09 | 1.00 | 1.05 | 1.15 |
| 4 | RYGB 2015-2019 | Robotic approach | 16,242 | 14665.98 | 0.98 | 2.04 | 2.29 |
| 4 | RYGB 2020-2024 | Laparoscopic approach | 150,820 | 149696.99 | 1.00 | 1.13 | 1.18 |
| 4 | RYGB 2020-2024 | Robotic approach | 70,786 | 67012.83 | 0.99 | 2.03 | 2.33 |
| 4 | BPD-DS 2015-2019 | Laparoscopic approach | 5,471 | 5429.45 | 1.00 | 1.30 | 1.45 |
| 4 | BPD-DS 2015-2019 | Robotic approach | 1,489 | 1370.02 | 0.95 | 1.61 | 2.58 |
| 4 | BPD-DS 2020-2024 | Laparoscopic approach | 5,953 | 5746.45 | 0.99 | 1.69 | 1.85 |
| 4 | BPD-DS 2020-2024 | Robotic approach | 3,794 | 3505.13 | 0.97 | 1.86 | 2.38 |
| 5 | RYGB 2015-2019 | Laparoscopic approach | 156,600 | 156472.98 | 1.00 | 1.05 | 1.15 |
| 5 | RYGB 2015-2019 | Robotic approach | 16,242 | 14677.14 | 0.98 | 2.04 | 2.28 |
| 5 | RYGB 2020-2024 | Laparoscopic approach | 150,820 | 149695.92 | 1.00 | 1.13 | 1.18 |
| 5 | RYGB 2020-2024 | Robotic approach | 70,786 | 67015.58 | 0.99 | 2.02 | 2.33 |
| 5 | BPD-DS 2015-2019 | Laparoscopic approach | 5,471 | 5430.00 | 1.00 | 1.29 | 1.51 |
| 5 | BPD-DS 2015-2019 | Robotic approach | 1,489 | 1372.08 | 0.94 | 1.61 | 2.18 |
| 5 | BPD-DS 2020-2024 | Laparoscopic approach | 5,953 | 5745.04 | 0.99 | 1.68 | 1.88 |
| 5 | BPD-DS 2020-2024 | Robotic approach | 3,794 | 3503.90 | 0.97 | 1.86 | 2.49 |
| Abbreviations: BPD-DS, biliopancreatic diversion with duodenal switch; RYGB, Roux-en-Y gastric bypass; sIPTW, stabilized inverse probability of treatment weighting. Effective sample size was calculated as (sum of weights)^2 divided by the sum of squared weights. Weights were estimated separately within each procedure-period stratum. | | | | | | | |
|  | | | | | | | |

**Table S4:** Complete-case covariate balance before and after stratum-specific stabilized inverse probability of treatment weighting

|  | **RYGB 2015-2019** | | **RYGB 2020-2024** | | **BPD-DS 2015-2019** | | **BPD-DS 2020-2024** | |
| --- | --- | --- | --- | --- | --- | --- | --- | --- |
| **Covariate** | **Unadjusted aSMD** | **sIPTW aSMD** | **Unadjusted aSMD** | **sIPTW aSMD** | **Unadjusted aSMD** | **sIPTW aSMD** | **Unadjusted aSMD** | **sIPTW aSMD** |
| Age | 0.09 | 0.04 | 0.03 | 0.02 | 0.10 | 0.04 | 0.08 | 0.00 |
| Female | 0.00 | 0.01 | 0.01 | 0.00 | 0.09 | 0.04 | 0.03 | 0.05 |
| Body mass index | 0.07 | 0.04 | 0.02 | 0.01 | 0.11 | 0.04 | 0.33 | 0.08 |
| White race | 0.00 | 0.01 | 0.04 | 0.02 | 0.29 | 0.09 | 0.23 | 0.05 |
| Black or African American race | 0.02 | 0.03 | 0.04 | 0.02 | 0.29 | 0.08 | 0.32 | 0.07 |
| Dependent functional status | 0.06 | 0.02 | 0.00 | 0.00 | 0.09 | 0.08 | 0.01 | 0.02 |
| ASA class III or higher | 0.04 | 0.04 | 0.03 | 0.02 | 0.10 | 0.09 | 0.01 | 0.03 |
| Albumin | 0.03 | 0.01 | 0.09 | 0.02 | 0.01 | 0.04 | 0.01 | 0.04 |
| Hematocrit | 0.04 | 0.04 | 0.03 | 0.01 | 0.16 | 0.06 | 0.28 | 0.07 |
| Diabetes | 0.05 | 0.03 | 0.02 | 0.01 | 0.03 | 0.00 | 0.00 | 0.04 |
| Hypertension | 0.04 | 0.02 | 0.05 | 0.02 | 0.01 | 0.01 | 0.08 | 0.06 |
| Hyperlipidemia | 0.05 | 0.02 | 0.04 | 0.03 | 0.04 | 0.01 | 0.01 | 0.04 |
| GERD | 0.11 | 0.05 | 0.08 | 0.03 | 0.29 | 0.10 | 0.01 | 0.01 |
| Therapeutic anticoagulation | 0.04 | 0.03 | 0.01 | 0.00 | 0.02 | 0.01 | 0.02 | 0.01 |
| COPD | 0.06 | 0.03 | 0.02 | 0.01 | 0.06 | 0.05 | 0.00 | 0.01 |
| History of pulmonary embolism | 0.00 | 0.00 | 0.01 | 0.01 | 0.01 | 0.01 | 0.01 | 0.03 |
| Sleep apnea | 0.04 | 0.03 | 0.00 | 0.01 | 0.07 | 0.04 | 0.03 | 0.04 |
| History of myocardial infarction | 0.00 | 0.01 | 0.00 | 0.01 | 0.02 | 0.04 | 0.03 | 0.03 |
| PCI | 0.03 | 0.01 | 0.00 | 0.01 | 0.06 | 0.05 | 0.03 | 0.01 |
| Previous cardiac surgery | 0.01 | 0.00 | 0.00 | 0.01 | 0.06 | 0.05 | 0.01 | 0.01 |
| History of deep vein thrombosis | 0.01 | 0.02 | 0.01 | 0.01 | 0.00 | 0.02 | 0.01 | 0.01 |
| Venous stasis | 0.01 | 0.01 | 0.03 | 0.02 | 0.09 | 0.09 | 0.02 | 0.03 |
| Dialysis | 0.01 | 0.02 | 0.01 | 0.00 | 0.02 | 0.00 | 0.00 | 0.00 |
| Renal insufficiency | 0.01 | 0.01 | 0.01 | 0.01 | 0.04 | 0.03 | 0.04 | 0.05 |
| Previous surgery | 0.02 | 0.03 | 0.02 | 0.01 | 0.12 | 0.09 | 0.05 | 0.05 |
| Current smoker | 0.04 | 0.03 | 0.00 | 0.00 | 0.01 | 0.00 | 0.02 | 0.01 |
| Drain placed | 0.35 | 0.06 | 0.33 | 0.04 | 0.33 | 0.04 | 0.10 | 0.02 |
| Values are absolute standardized mean differences before and after stratum-specific stabilized inverse probability of treatment weighting in the complete-case cohort. Propensity-score models were fitted separately within each procedure-period stratum and excluded conversion to open surgery. Values <0.10 were considered indicative of acceptable covariate balance. Abbreviations: aSMD, absolute standardized mean difference; ASA, American Society of Anesthesiologists; BPD-DS, biliopancreatic diversion with duodenal switch; RYGB, Roux-en-Y gastric bypass; sIPTW, stabilized inverse probability of treatment weighting. | | | | | | | | |

**Table S5:** Complete-case cell sizes for grouped 30-day endpoints and operative length across procedure-period strata

|  | **RYGB 2015-2019** | | **RYGB 2020-2024** | | **BPD-DS 2015-2019** | | **BPD-DS 2020-2024** | |
| --- | --- | --- | --- | --- | --- | --- | --- | --- |
| **Outcome** | **Robotic** | **Laparoscopic** | **Robotic** | **Laparoscopic** | **Robotic** | **Laparoscopic** | **Robotic** | **Laparoscopic** |
| Mortality | 12/11,468 (0.1) | 160/103,361 (0.2) | 71/54,024 (0.1) | 108/107,532 (0.1) | 0/1,056 (0.0) | 13/3,702 (0.4) | 8/2,658 (0.3) | 4/4,237 (0.1) |
| Prolonged index stay | 1,573/11,468 (15.1) | 14,825/103,361 (14.3) | 3,828/54,019 (7.5) | 8,466/107,522 (7.8) | 507/1,056 (41.6) | 755/3,702 (21.5) | 301/2,656 (10.7) | 509/4,236 (13.0) |
| Readmission | 797/11,468 (6.9) | 5,985/103,361 (5.8) | 2,888/54,024 (5.4) | 4,958/107,532 (4.6) | 84/1,056 (8.0) | 229/3,702 (6.3) | 136/2,658 (5.1) | 188/4,237 (4.5) |
| Reoperation | 281/11,468 (2.4) | 2,350/103,361 (2.3) | 959/54,024 (1.8) | 1,850/107,532 (1.7) | 43/1,056 (4.0) | 128/3,702 (3.5) | 53/2,658 (2.1) | 109/4,237 (2.6) |
| Intervention | 272/11,468 (2.4) | 2,377/103,361 (2.3) | 768/54,024 (1.4) | 1,515/107,532 (1.4) | 35/1,056 (3.5) | 80/3,702 (2.2) | 32/2,658 (1.2) | 55/4,237 (1.3) |
| Serious complications | 233/11,468 (2.0) | 2,645/103,361 (2.6) | 1,027/54,024 (1.9) | 2,390/107,532 (2.2) | 44/1,056 (4.0) | 124/3,702 (3.4) | 69/2,658 (2.7) | 112/4,237 (2.7) |
| Operative length | 155.37 ± 62.97 | 115.92 ± 52.67 | 144.84 ± 58.04 | 118.65 ± 54.10 | 209.01 ± 69.37 | 130.19 ± 67.64 | 161.58 ± 67.51 | 126.41 ± 62.08 |
| Values are from the complete-case weighted cohort. Categorical outcomes are presented as events/N with weighted percentages in parentheses; percent signs are omitted from table cells. Operative length is presented as weighted mean ± weighted SD. Prolonged index stay was defined as index hospital stay >=3 days. Serious complications were defined as a composite of postoperative sepsis, septic shock, organ-space surgical-site infection, deep incisional surgical-site infection, transfusion or bleeding, pulmonary embolism, venous thrombosis requiring therapy, pneumonia, ventilator use, acute renal failure, progressive renal insufficiency, cardiac arrest requiring CPR, myocardial infarction, and stroke. Abbreviations: BPD-DS, biliopancreatic diversion with duodenal switch; RYGB, Roux-en-Y gastric bypass; SD, standard deviation; sIPTW, stabilized inverse probability of treatment weighting. | | | | | | | | |

**Table S6:** Complete-case stratum-specific propensity-weighted associations between robotic and laparoscopic approaches and grouped 30-day endpoints and operative length

|  | **RYGB 2015-2019** | | | **RYGB 2020-2024** | | | **BPD-DS 2015-2019** | | | **BPD-DS 2020-2024** | | |
| --- | --- | --- | --- | --- | --- | --- | --- | --- | --- | --- | --- | --- |
| **Outcome** | **OR/MD (95% CI)** | **Unadjusted p** | **BH-adjusted p** | **OR/MD (95% CI)** | **Unadjusted p** | **BH-adjusted p** | **OR/MD (95% CI)** | **Unadjusted p** | **BH-adjusted p** | **OR/MD (95% CI)** | **Unadjusted p** | **BH-adjusted p** |
| Mortality | 0.61 (0.34-1.12) | 0.110 | 0.171 | 1.29 (0.95-1.76) | 0.106 | 0.171 | 0.00 (0.00-0.00) | <0.001 | <0.001 | 3.31 (0.95-11.58) | 0.061 | 0.107 |
| Prolonged index stay | 1.07 (1.01-1.13) | 0.029 | 0.062 | 0.96 (0.92-1.00) | 0.060 | 0.107 | 2.60 (2.24-3.02) | <0.001 | <0.001 | 0.80 (0.68-0.93) | 0.005 | 0.012 |
| Readmission | 1.20 (1.11-1.30) | <0.001 | <0.001 | 1.16 (1.11-1.22) | <0.001 | <0.001 | 1.31 (0.99-1.72) | 0.055 | 0.107 | 1.13 (0.89-1.42) | 0.311 | 0.415 |
| Reoperation | 1.06 (0.93-1.21) | 0.351 | 0.447 | 1.05 (0.96-1.13) | 0.274 | 0.384 | 1.14 (0.79-1.66) | 0.475 | 0.554 | 0.80 (0.57-1.13) | 0.202 | 0.298 |
| Intervention | 1.04 (0.91-1.19) | 0.540 | 0.605 | 1.01 (0.93-1.11) | 0.752 | 0.780 | 1.63 (1.07-2.49) | 0.022 | 0.052 | 0.91 (0.58-1.43) | 0.680 | 0.733 |
| Serious complications | 0.78 (0.68-0.90) | <0.001 | 0.002 | 0.86 (0.80-0.93) | <0.001 | <0.001 | 1.18 (0.82-1.71) | 0.370 | 0.450 | 0.97 (0.71-1.33) | 0.861 | 0.861 |
| Operative length | 39.45 (38.13-40.77) | <0.001 | <0.001 | 26.19 (25.57-26.80) | <0.001 | <0.001 | 78.82 (73.75-83.89) | <0.001 | <0.001 | 35.17 (31.85-38.49) | <0.001 | <0.001 |
| Values are presented as odds ratios with 95% confidence intervals for binary endpoints and mean differences with 95% confidence intervals for operative length. All estimates compare robotic with laparoscopic approaches within each procedure-period stratum in the complete-case cohort. Propensity-score models were fitted separately within each procedure-period stratum and excluded conversion to open surgery. Prolonged index stay was defined as index hospital stay >=3 days. Serious complications were defined as a composite of postoperative sepsis, septic shock, organ-space surgical-site infection, deep incisional surgical-site infection, transfusion or bleeding, pulmonary embolism, venous thrombosis requiring therapy, pneumonia, ventilator use, acute renal failure, progressive renal insufficiency, cardiac arrest requiring CPR, myocardial infarction, and stroke. Benjamini-Hochberg adjusted p values were calculated across the full family of 28 procedure-period endpoint comparisons. Abbreviations: BH, Benjamini-Hochberg; BPD-DS, biliopancreatic diversion with duodenal switch; CI, confidence interval; MD, mean difference; OR, odds ratio; RYGB, Roux-en-Y gastric bypass. | | | | | | | | | | | | |

**Figure S2:** Covariate balance before and after stabilized inverse probability of treatment weighting (sIPTW) in the complete-case cohort

 **Figure S3:** Covariate balance before and after stabilized inverse probability of treatment weighting (sIPTW) in imputed dataset 2.

 **Figure S4:** Covariate balance before and after stabilized inverse probability of treatment weighting (sIPTW) in imputed dataset 3

 **Figure S5:** Covariate balance before and after stabilized inverse probability of treatment weighting (sIPTW) in imputed dataset 4

 **Figure S6:** Covariate balance before and after stabilized inverse probability of treatment weighting (sIPTW) in imputed dataset 5.
